# Owls’ Responses to Environmental Challenges

**DOI:** 10.3390/ani14060880

**Published:** 2024-03-13

**Authors:** Tapio Solonen

**Affiliations:** Luontotutkimus Solonen Oy, Neitsytsaarentie 7b B 147, FI-00960 Helsinki, Finland; tapio.solonen@pp.inet.fi

Owls are a group of predatory birds characterized by their largely nocturnal way of life [1,2]. Their food supply, in particular small microtines, often fluctuates a lot annually. Some species of owls prefer old forests that provide tree hollows or other suitable nest sites, while other species occupy open habitats that provide, at least from time to time, plenty of suitable prey. Owls have two main types of lifestyles: some species are strictly stationary, while some others are nomads, continuously searching for areas with rich food supplies in which to settle down. As distributions shift and population densities change, new species interactions result in changes in competition and predation. Due to their wide habitat spectrum and their position near the top of their food chains, owls have the potential to serve as indicators of various environmental changes [3].

This Special Issue focuses on the adaptability of owls to various kinds of environmental changes, including habitat alterations and climate change [4,5,6,7,8,9,10]. Both direct and indirect effects of climate change on the habitat and food availability of owl populations are considered. Various populations of the Tawny Owl *Strix aluco* [4,7,8] as well as some other species of owls from Europe [5,6,9] and North America [10] serve as examples.

Urbanization causes major land use changes around the world, with vast effects on wildlife [11,12]. Due to urbanization, natural and seminatural habitats are being altered and destroyed worldwide, leading to a decrease in species abundance and richness. Nevertheless, the Tawny Owl has successfully colonized novel urban habitats [4]. At the local scale, changes in both forests and urban land covers have affected its abundance, with medium forested areas being its current optimal habitat. However, even when an urbanized area has minimal forest structures, the Tawny Owl can exploit the habitat. At the landscape scale, it prefers smaller urban towns to cities. Thus, the current tendency toward green cities would benefit Tawny Owl populations. In urban landscapes, the size of owl territories may be smaller than in rural landscapes [5], and the compositional differences between the territories in these two landscapes may have important consequences for other types of behaviors and possibly the reproductive output of Tawny Owls.

Wind power is useful for reducing greenhouse gas emissions, but their construction and operation might have negative effects on biodiversity [13,14]. For instance, wind farms and power line construction negatively affect territory occupancy in the vulnerable Norwegian populations of the Eurasian Eagle Owl *Bubo bubo* [6]. A study showed that the distances from their nest sites to disturbances were significantly shorter for territories they abandoned compared with territories where they stayed. Possible reasons for this decline might be the higher mortality caused by collisions with, desertion of, and avoidance of wind power areas. There may be indirect effects as well. For instance, reductions in prey species may force owls to abandon their territories.

Owls’ responses to fluctuating weather conditions may indicate the direction of climate change and its subsequent potential effects. The body conditions of vole-eater boreal species seem to vary largely according to both the weather conditions during the preceding winter and fluctuations in the vole populations [7]. The weather conditions during the winter that precedes breeding have pronounced effects on the food availability of Tawny Owls near the northern limit of the species’ distribution range. Deep snow cover protects voles through winter until spring better than a minor amount of snow, and frequent temperature fluctuations around the freezing point in early spring make voles more easily available for owls that are preparing to breed. Due to the efficient use of alternative prey, the effects of fluctuating vole populations on the body condition of Tawny Owls are, in general, only moderate. At the other end of the distribution spectrum, Tawny Owls are more susceptible to climate than land cover transformations in Israel [8]. The number of fledglings increases with precipitation in rural settings but is adversely affected by extreme temperatures. The Tawny Owl can adapt to rural–agricultural and urban environments but cannot escape the impacts of climate change.

Knowledge of their current and future geographic distributions is essential for conserving endangered species. Spatial distribution models show that, compared with the current areas, areas highly suitable for the Boreal Owl *Aegolius funereus* with increase in the Balkan Peninsula in 2041–2060 [9]. However, during the next 20 years, the amount of highly suitable areas will reduce. For the Eurasian Pygmy Owl *Glaucidium passerinum*, highly suitable areas are forecasted to decrease during 2041–2060 but to then increase during 2061–2080. The conservation and preservation of areas with potential for the distribution and refugia of Boreal and Eurasian Pygmy Owls are therefore important in the face of climate change. Thus, the forecasting of vegetation-type shifts may prove essential in anticipating and mitigating the impacts of future climate change on bird populations [10]. For instance, high exposure to climate change is suggested to impact the upper-elevation forest owls of semi-arid Southwestern North America, and long-distance migration and low natal philopatry may prove important to some montane owl populations in adapting to the regional loss of habitat.

In summary, this Special Issue presents a short, up-to-date overview of owls’ responses to various environmental challenges. Owls seem to adapt flexibly to many environmental changes. However, they need habitats that fulfil their food and nesting requirements. Thus, the continuous availability of such habitats depends largely on human activities.
